# Increasing access to mental health supports for healthcare workers during the COVID-19 pandemic and beyond through a novel coaching program

**DOI:** 10.3389/fpsyt.2022.1073639

**Published:** 2022-12-22

**Authors:** Kimberly T. Arnold, Emily M. Becker-Haimes, Katherine Wislocki, Lisa Bellini, Cecilia Livesey, Kelley Kugler, Michal Weiss, Courtney Benjamin Wolk

**Affiliations:** ^1^Department of Family Medicine and Community Health, University of Pennsylvania Perelman School of Medicine, Philadelphia, PA, United States; ^2^Department of Psychiatry, University of Pennsylvania Perelman School of Medicine, Philadelphia, PA, United States; ^3^Department of Medicine, University of Pennsylvania Perelman School of Medicine, Philadelphia, PA, United States

**Keywords:** COVID-19, resiliency, wellness, healthcare workers, Psychological First Aid

## Abstract

The COVID-19 pandemic led to heightened anxiety, distress, and burnout among healthcare workers and faculty in academic medicine. Penn Medicine launched Coping First Aid (CFA) in March 2020 in response to the pandemic. Informed by Psychological First Aid principles and therapeutic micro skills, CFA was designed as a tele-mental healthcare service for health system employees and their families delivered by trained lay volunteer coaches under the supervision of licensed mental health clinicians. We present an overview of the model, feasibility and utilization data, and preliminary implementation and effectiveness outcomes based on cross sectional coach (*n* = 22) and client (*n* = 57) self-report surveys with a subset of program users in the first year. A total of 44 individuals completed training and were certified to coach. Over the first 24 months of the program, 513 sessions occurred with 273 clients (119 sessions were no-shows or canceled). Follow-up appointments were recommended in 52.6% (*n* = 270) of sessions and 21.2% (*n* = 109) of clients were referred for professional mental health care. Client survey respondents reported CFA was helpful; 60% were very or extremely satisfied, and 74% indicated they would recommend the program. Our preliminary findings suggest that CFA was feasible to implement and most clients found the service beneficial. CFA provides a model for rapidly developing and scaling mental health supports during and beyond the pandemic.

## Introduction

The COVID-19 pandemic heightened anxiety, distress, and burnout among healthcare workers (HCWs), faculty, and trainees in academic medical centers (1). This includes fear of exposure to COVID-19, transmission of the virus to loved ones, a high degree of uncertainty in the work environment, and lack of support for personal concerns—all while juggling demanding careers (2). The extant literature suggests that up to 50% of workers directly impacted by a pandemic may exhibit persistent, clinically significant distress (3). Emerging research from the COVID-19 pandemic suggests a similar or worse trajectory (2, 4). Facilitating access to effective treatment and psychosocial support is critical to mitigating negative impacts on mental health, minimizing distress, and reducing burnout (5, 6).

Easily accessible, highly relevant, and demand-oriented services that are designed for efficient implementation and rapid scale-up are needed (7, 8). This Brief Research Report describes a novel clinical service designed to provide such support and presents 24 months program evaluation data of implementation (feasibility, acceptability) and preliminary effectiveness outcomes.

## Materials and methods

### Program description

To address HCWs’ needs during the COVID-19 pandemic, we developed and implemented the Coping First Aid (CFA) program at Penn Medicine (developed in March and launched in April of 2020). Penn Medicine employs more than 42,000 individuals and includes six acute-care hospitals and hundreds of outpatient locations throughout the greater Philadelphia region. CFA is delivered by lay health volunteers referred to as *CFA Coaches*. CFA is hosted on a digital platform (Penn COBALT), which provides a suite of behavioral health and wellness supports to those affiliated with Penn Medicine. CFA is grounded in principles of Psychological First Aid (9) and therapeutic micro skills and adapted specifically for COVID-19 (i.e., adapted to be delivered *via* telehealth and to incorporate COVID-19 specific examples to illustrate principles and enhance relevance during the pandemic). Coaches provide non-intrusive practical care, assess needs and concerns, and focus on listening and comforting those in need. Coaches are also trained to help link HCWs to needed services and supports and engage in structured, collaborative problem-solving. A call for volunteers was initially put out by health system administrators at the time of program launch; any health system employee or trainee was permitted to apply to volunteer to coach, except for already licensed mental health clinicians.

The implementation model was designed to be exclusively virtual. The CFA team developed an asynchronous 3 h webinar training, a system for potential coaches to demonstrate their skills using video-recorded standardized role-plays, provided session summary sheets and checklists for coaches to use to guide their delivery of session content, and a series of tip sheets for CFA Coaches to share with HCWs (e.g., telehealth best practices, sleep hygiene, practicing self-care). The training was developed as a webinar that was hosted on a password protected website that coaches could complete on their own time after being accepted into the program as a coach trainee. After completing this initial training and before coaching, coaches completed a structured role play using an established fidelity measure (10) adapted for COVID-19. Role-plays were video-recorded and rated by psychologists and trained graduate student coders who utilized a yes/no checklist coding process to denote whether core features of the model were delivered;(10) coaches also were provided written qualitative feedback about strengths and areas for growth. Once certified, coaches set weekly 60 min timeslots with their availability to meet with clients *via* secure telemedicine platform. Coaches receive weekly group supervision with a mental health professional to guide their care provision, with clear escalation protocols to use in an emergency (e.g., should suicidality be endorsed) to connect with a licensed mental health practitioner. Coaches also receive training in when and how to implement the escalation protocol and with respect to connecting individuals in need of higher levels of care to appropriate supports. Client information remains confidential to the extent allowable by law. Coaches complete session report forms that summarize pertinent information from each session using predefined categories, which supervisors review weekly.

### Program evaluation procedures

Administrative and program records and coach and client surveys were used to descriptively examine outcomes of interest. Procedures were reviewed and approved by the University of Pennsylvania Institutional Review Board (protocol #844318; see Supplemental material for copies of measures). Coach and client participants who completed the optional surveys provided informed consent before participating. Analyses were conducted in SPSS (11).

#### Administrative and program records

We obtained program utilization data through reports provided regularly by the technology support team overseeing the platform that hosted CFA for the first 24 months of the program (April 2020–March 2022). Therapeutic strategies employed by coaches were entered by coaches directly into predefined fields in REDCap, a secure platform in compliance with the Health Insurance Portability and Accountability Act (HIPAA) of 1996 to protect the privacy of health information, using a structured post-session report form immediately following each encounter.

#### Coach and client surveys

Coaches who completed CFA training and were still active coaches 1 year into the program (*n* = 38) and clients who scheduled one or more sessions and provided a valid email address at the time they booked the appointment (*n* = 166) were invited by email to participate in a one-time, brief, 5–10 min survey at the conclusion of the first year of program operation. Surveys included informed consent language on the first page; proceeding to the survey constituted consent. For completing the survey, client and coach participants each had the option to be entered into separate lotteries to win one of two $25 gift cards. Client surveys were anonymous; no identifying information was collected unless clients opted to provide their emails to be entered into the incentive lottery. Coaches were informed that their survey responses may be linked to other administrative data. Survey invitations were emailed between December 2020 and January 2021 *via* REDCap.

The coach survey included 13 questions about perceived acceptability and effectiveness. Coaches also completed the Acceptability of Intervention Measure (AIM), (12) an established, validated measure of intervention acceptability (Cronbach’s α = 0.87), about the CFA program. The client survey included 18 items that assessed client background, reasons for seeking support, and perceived effectiveness of and satisfaction with CFA. The full surveys are available as a supplemental file. Survey data reflect the final outcomes of this one-time cross-sectional survey of clients and coaches.

## Results

### Reach

At the time of analysis, CFA had been operating for 24 months in a health system that employs tens of thousands of employees. During this period, 632 client sessions were scheduled, 513 sessions occurred, and 119 clients either no-showed or canceled prior to the appointment. Of the 513 sessions that occurred, the mean length of sessions reported was 46.68 (SD = 15.56) minutes. Follow-up appointments were recommended in 53.2% of completed sessions (to be booked by the client at their discretion) and 45.8% (*n* = 109) of clients were referred for higher-level, professional mental health care.

### Feasibility and acceptability

An initial call for volunteers sent to the University of Pennsylvania Health System (i.e., Penn Medicine) at program launch resulted in 119 volunteers. Of these, 100 completed the initial training webinar. Only 44 (44%) of the 100 individuals trained went on to complete role-plays and were certified to begin coaching. Only three coaches did not achieve competency on their first role-play; all were certified after their second attempt. Regarding coach retention, of certified coaches, 34 delivered at least 1 session, 15 were still volunteering after 1 year and nine were still volunteering after 2 years.

### Individual concerns discussed in sessions

Clients presented for coaching sessions to discuss varying concerns. Most commonly noted issues were emotional concerns (55.9% of clients), physical symptoms (25.1% of clients), and other specific concerns (e.g., COVID-19 topics, financial concerns, family/relationship stress, workplace issues; 24.4% of clients). Less commonly, clients presented with cognitive concerns (12.7%) and behavioral concerns (9.0%). See Table 1 for list of client concerns documented during coaching sessions.

**TABLE 1:** Client concerns documented in completed coaching sessions (*N* = 513).

	*n* (%)
Emotional concerns	
Anxiety, fearful	165 (32.2%)
Sadness, tearful	93 (18.1%)
Feelings of guilt or shame	60 (11.7%)
Acute stress reactions	47 (9.2%)
Despair, hopeless	32 (6.2%)
Irritability, anger	28 (5.5%)
Acute grief reactions	15 (2.9%)
Feeling numb or disconnected	15 (2.9%)
Other emotional concern	32 (6.2%)
**Physical concerns**	
Sleep difficulties	78 (15.2%)
Fatigue or exhaustion	43 (8.4%)
Difficulty eating	16 (3.1%)
Stomachaches	14 (2.7%)
Headac hes	11 (2.2%)
Worsening health condition	5 (1.0%)
Other physical concern	13 (2.5%)
**Behavioral concerns**	
Isolation or withdrawal	24 (4.7%)
Maladaptive coping	12 (2.3%)
Drug or alcohol use	3 (0.6%)
Separation anxiety	2 (0.4%)
Regressive behavior	1 (0.2%)
Other behavioral concern	8 (1.6%)
**Cognitive concerns**	
Difficulty concentrating	34 (6.6%)
Difficulty making decisions	22 (4.3%)
Intrusive thoughts or images	15 (2.9%)
Difficulty remembering	1 (0.2%)
Distressing dreams or nightmares	1 (0.2%)
Other cognitive concern	11 (2.1%)
**Other concerns**	
Past or pre-existing trauma/psychological problems/substance abuse problems	37 (7.2%)
Living arrangements	27 (5.3%)
Concerns about child/adolescent	18 (3.5%)
Financial concerns	15 (2.9%)
Spiritual concerns	12 (2.3%)
Loved one(s) diagnosed or hospitalized with COVID-19	6 (1.2%)
Has been diagnosed with COVID-19	5 (1.0%)
Lost job or school	3 (0.6%)
At risk of losing own life	1 (0.2%)
Medication stabilization	1 (0.2%)
Other concerns, not otherwise specified	30 (5.8%)

### Intervention techniques used

In 63.4% (*n* = 325) of sessions that occurred, coaches took steps to ensure the safety and comfort of the client (e.g., ensured client’s physical safety, asked about immediate needs). Coaches engaged in information gathering (e.g., assessed client concerns about the pandemic, assessed client’s social support network) in 60.6% (*n* = 311) of sessions, engaged in stabilization (e.g., used grounding or relaxation techniques) in 21.2% (*n* = 109) of sessions, provided practical assistance (e.g., helped client to develop an action plan) in 91.3% (*n* = 469) of sessions, and facilitated connection with social supports (e.g., helped problem solve obtaining support) in 65.7% (*n* = 337) of sessions.

### Coach and client surveys

Surveys to assess preliminary outcomes were sent to coaches (*n* = 38) and clients (*n* = 166); five client emails were returned as undeliverable.

#### Coach survey results

Coach survey respondents (*n* = 22) were a mean (SD) age of 40 (13), 68% female; 59% White, 9% Black, 9% Asian, 14% Multiracial or Other, and 0% Hispanic/Latinx. Of coaches who completed the satisfaction survey (*n* = 22), 54.5% of respondents strongly agreed (i.e., endorsed “very true for me”) that CFA training prepared them to provide coaching, and the remainder (45.5%) stated the program somewhat prepared them. Nearly all (86.4%) strongly agreed with statements that supervision prepared them for coaching, with the remainder (13.6%) indicating that this was somewhat true. Overall, 77.3% of coaches indicated strong agreement with the statement *“Overall, my experience with the CFA program is/was positive,”* 22.7% indicated that this was somewhat true for them, and no respondents indicated that this was not true. Average coach scores on the AIM also indicated high acceptability (M = 4.57, SD = 0.50). However, only 22.7% of coaches agreed that it was easy for them to make connections to mental health services for clients in need, and 68.2% said that this was only somewhat true for them.

#### Client survey results

Client survey respondents (*n* = 57) were a mean (SD) age of 38 (12); 75% female; 58% White, 12% Black, 16% Asian, 14% Multiracial or Other, and 7% Hispanic/Latinx. Clients surveyed at the end of the first program year reported seeking services for the program for a variety of reasons, including anxiety or distress related to COVID-19 (*n* = 11, 19%), anxiety or emotional distress related to other concerns (*n* = 31, 53.4%), difficulty coping at work (*n* = 2, 3.4%), difficulty coping in one’s personal life (*n* = 5, 8.6%), and other (*n* = 2, 3.4%); 4 (6.9%) stated that they were coping well overall and not experiencing emotional distress.

Satisfaction and perceived efficacy within this subsample were variable, although positive overall. Specifically, 59.6% (*n* = 34) of survey respondents reported that they were very or extremely satisfied with CFA, 15.8% (*n* = 9) were somewhat satisfied, 12.3% (*n* = 7) were only a little satisfied, and 9.8% (*n* = 5) were not at all satisfied. Most (*n* = 42, 73.7%) said that they would probably or definitely recommend CFA to a friend, 4 (7.0%) said they possibly would, and 9 (15.8%) said that they probably or definitely would not recommend CFA to a friend; see Table 2. Specific items rated positively by most respondents included satisfaction with how the coach listened (77.2%), assessed needs and concerns (64.9%), and provided resources (50.9%); most also agreed that the virtual platform worked well (52.6%). A minority reported that it was very true that CFA reduced their anxiety or emotional distress (35.1%) and helped them cope with challenges related to COVID-19 (24.6%).

**TABLE 2:** Client satisfaction and perceived efficacy of the Coping First Aid (CFA) program from client survey respondents (*N* = 57)^1^.

	Not at all true	Somewhat true	Very true
	*n* (%)	*n* (%)	*n* (%)
Coping First Aid reduced my general anxiety and/or emotional distress	9 (15.8)	26 (45.6)	20 (35.1)
Coping First Aid helped me cope with challenges related to COVID-19	12 (21.1)	29 (50.9)	14 (24.6)
My Coach assessed my needs and concerns	4 (7.0)	14 (24.6)	37 (64.9)
My Coach listened to me	3 (5.3%)	8 (14.0)	44 (77.2)
My Coach understood my concerns	5 (8.8)	11 (19.3)	39 (68.4)
My COVID Coach provided helpful resources	9 (15.8)	17 (29.8)	29 (50.9)
The virtual platform worked well for me	6 (10.5)	19 (33.3)	30 (52.6)

^1^Two respondents left these survey items blank, so percentages do not add to 100.

We examined whether there were differences in client satisfaction with respect to reason for seeking services (were clients seeking help for COVID-19 related concerns or not), client’s job position (clinical vs. non-clinical), and client demographics (age, female gender or not, Person of Color or not) to determine if there were major inequities in outcomes that required attention. We dichotomized satisfaction results as highly satisfied (very or extremely satisfied) versus not satisfied (somewhat, only a little, or not at all satisfied). Results indicated no differences on any examined variable (all *p*s > 0.05; see Table 3).

**TABLE 3:** Chi-square analysis of client survey respondent satisfaction and hypothesized background factors^1^.

Client factors	Not satisfied	Highly satisfied	Test statistic	*P*
Sign-up reason, *n*	–	–	*X*^2^ = 0.582	0.446
COVID-related	12	15	–	–
Not COVID-related	10	19	–	–
Position, *n*	–	–	*X*^2^ = 0.194	0.660
Clinical facing	10	17	–	–
Non-clinical facing	12	16	–	–
Racial identity, *n*	–	–	*X*^2^ = 0.013	0.911
Person of color	9	13	–	–
Not person of color	13	20	–	–
Gender, *n*	–	–	*X*^2^ = 1.360	0.244
Female	15	27	–	–
Non-female	7	6	–	–
Client age, M (SD)	37.24 (12.32)	38.64 (12.27)	t (52) = –0.408	0.685

^1^Due to missing data, sample sizes for each candidate predictor of interest were as follows: “sign-up reason” *n* = 56; “client age” *n* = 54; all other analyses sample size n = 55.

## Discussion

We launched CFA at Penn Medicine early in the COVID-19 pandemic as an accessible, free support service for those experiencing distress or difficulty coping related to the pandemic. The program was designed and implemented quickly, given the pandemic’s urgency, and leveraged lay coaches to preserve limited mental health resources for those with highest need. Coach volunteers were quickly assembled, trained, on-boarded, and deployed. Our preliminary program evaluation of CFA suggests most coaches and clients found the program acceptable and that it was feasible to implement; however, more work is needed to reach those who may benefit. Our coach survey results also highlighted the importance of regular supervision from licensed mental health professionals for lay coaches to feel adequately supported in providing wellness services.

Clients presented to CFA with a range of emotional and behavioral concerns. Given that the program was developed to meet need during the COVID-19 pandemic, it was surprising that few clients presented with anxiety or other emotional concerns related to contracting or spreading COVID-19. Rather, many clients brought general personal and professional concerns to coaching calls, some of which likely were exacerbated by the pandemic. This highlights that programs like CFA may have utility post-pandemic in supporting and sustaining the healthcare workforce.

Our evaluation also underscored the difficulties HCWs experienced when trying to connect with mental health care when referred by our coaching program. Even with their training and in a system with an established infrastructure for connecting employees to professional mental health services, CFA coaches reported struggling to connect clients in need with mental health professionals. The COVID-19 pandemic has exacerbated the need for mental health care and further exposed pre-existing mental health workforce shortages (13). To be optimally successful, lay coach-delivered programs like CFA ideally should be embedded within infrastructures that can support connecting individuals with higher levels of care. Lay coaches also should be knowledgeable of available resources and well-supported throughout the process of connecting clients to professional services.

Only a small fraction of eligible employees used CFA. This suggests that future work to market and disseminate this program is needed. Health systems that have developed emotional support programs during the pandemic should attend to utilization rates and consider shifting their efforts from developing content and programing to dissemination at this stage.

While strengths of CFA include the reliance on lay coaches and free nature of the service, these strengths raise concerns for the long-term sustainability of this and similar programs, as there is no billable revenue to support the program’s infrastructure and administrative costs. Future attention must be paid to developing long-term implementation and sustainment plans for continuing to support programs that are meeting employees’ mental health needs. Given that the mental health workforce is finite and limited, and that the need for services has only increased during the pandemic, (13) optimizing programs like CFA that leverage lay coaches may be helpful (8).

Our study utilized more than 500 administrative records of coaching encounters. This is a strength given the limited data included in many previous studies of emotional supports for healthcare workers (8). Nonetheless, the coach and client surveys were limited by small sample sizes and we have no control group. To maximize meaning and generalizability, more research is needed with larger samples and randomized designs. Previous research and our feasibility data suggest that lay coaches are promising for increasing access to support in a mental health system taxed by workforce shortages, supporting the importance of further research in this area.

Based on our experience, program evaluation data to date, and preliminary survey results, we recommend that organizations interested in implementing programs like CFA take the following steps:(7) (1) Establish a multidisciplinary implementation team to guide implementation; (2) Identify a coaching workforce (e.g., staff volunteers, mental health clinicians-in-training); (3) Plan for dissemination early by engaging end users about how best to reach them and meet their needs; (4) Develop a coach training and supervision plan and compile relevant materials/resources; (5) Define program confidentiality for employees and train coaches how to communicate this to clients; (6) Develop crisis protocols; (7) Make the program available to employees at a variety of times; (8) Disseminate information about the program clearly and often, using different modalities; and (9) Implement quality assurance practices and routine program evaluation, and iterate on the model and dissemination approach as needed.

## Data availability statement

The raw data supporting the conclusions of this article will be made available by the authors upon reasonable request.

## Ethics statement

The studies involving human participants were reviewed and approved by University of Pennsylvania Institutional Review Board. The patients/participants provided their informed consent to participate in this study.

## Author contributions

KA, EB-H, and CW: study conception and design and draft manuscript preparation. KA, KW, and MW: data collection. EB-H, KW, and MW: analysis and interpretation of results. All authors reviewed the results and approved the final version of the manuscript.
